# Serum indices based on creatinine and cystatin C predict mortality in patients with non-dialysis chronic kidney disease

**DOI:** 10.1038/s41598-021-96447-9

**Published:** 2021-08-19

**Authors:** Yu-Li Lin, I-Chen Chang, Hung-Hsiang Liou, Chih-Hsien Wang, Yu-Hsien Lai, Chiu-Huang Kuo, Bang-Gee Hsu

**Affiliations:** 1 Division of Nephrology, Hualien Tzu Chi Hospital, Buddhist Tzu Chi Medical Foundation, Hualien, 97004 Taiwan; 2 School of Medicine, Tzu Chi University, Hualien, 97004 Taiwan; 3 Division of Nephrology, Department of Internal Medicine, Hsin-Jen Hospital, New Taipei City, 24243 Taiwan

**Keywords:** Kidney diseases, Prognostic markers

## Abstract

Serum indices based on creatinine and cystatin C, including creatinine/cystatin C ratio (Cr/CysC), ratio and difference of estimated glomerular filtration rate (eGFR) based on cystatin C and creatinine (eGFRcys/eGFRcre and eGFR_Diff_), and serum creatinine × eGFRcys, are recently identified serum markers for sarcopenia. We aimed to evaluate the association between these serum indices and mortality in patients with chronic kidney disease (CKD). A single-center retrospective cohort study included 1141 adult patients with stage 1–5 CKD between 2016 and 2018. Basic characteristics, comorbidities, laboratory parameters, and serum creatinine and cystatin C values were obtained. Patients were followed up until death, dialysis, transfer to another hospital, or end of the study. The median age (interquartile range) of our participants was 71 (62–81) years. During a median follow-up of 39 months, 116 (10.2%) patients died. Compared to the survivor group, Cr/CysC, eGFRcys/eGFRcre, eGFR_Diff_, and Cr × eGFRcys were all lower in the non-survivors (*p* < 0.001 for all). The receiver operating characteristic curves of serum indices for predicting mortality showed that all four indices had significant discriminative power. Based on the Cox proportional hazard models, lower values of four serum indices, both as continuous and categorical variables, independently predicted mortality. Our findings suggest that low serum indices of Cr/CysC, eGFRcys/eGFRcre, eGFRDiff, and Cr × eGFRcys are independent indicators of mortality in patients with non-dialysis CKD.

## Introduction

Chronic kidney disease (CKD) is highly prevalent worldwide and represents a major public health problem^1^. Premature aging is a hallmark in CKD and metabolic derangements during the course of CKD adversely affect multiple organ systems, including skeletal muscle^2^. Compared with general senior population, patients with CKD are at higher risk for sarcopenia^3^, which is closely associated with increased mortality^4,5^.

Serum indices based on creatinine and cystatin C, two commonly used indicators of renal function, are recently identified as serum markers for sarcopenia. During muscle wasting, skeletal muscle loss is accompanied by reduced serum creatinine, which is released from skeletal muscle mass whereas serum cystatin C, which is produced by all nucleated cells, is relatively less affected^6,7^. Thus, low serum creatinine/cystatin C ratio (Cr/CysC) is recently identified as a marker for low skeletal muscle mass. Alternatively, in sarcopenic patients, estimated glomerular filtration rate (eGFR) calculated using serum creatinine (eGFRcre) can be overestimated compared to that calculated using cystatin C (eGFRcys), resulting in low ratios of eGFRcys/eGFRcre and wide differences between eGFRcys and eGFRcre (eGFR_Diff_). Accumulating evidence suggests that Cr/CysC, eGFRcys/eGFRcre, and eGFR_Diff_ may serve as useful surrogate markers for sarcopenia in different populations and that these indices are also associated with clinical outcomes^8–21^. Particularly, in patients with non-dialysis CKD, we previously demonstrated that Cr/CysC was correlated with both skeletal muscle mass and handgrip strength^19^. However, the association of these indices with mortality remains unexplored in patients with non-dialysis CKD.

Twenty-four-hour urine creatinine excretion, which is contributed primarily from skeletal muscle mass amount in steady renal function, is a known reliable urinary marker for sarcopenia in both the general and CKD populations^22–24^ and can be estimated by multiplying serum creatinine with 24-h creatinine clearance. eGFRcys, but not eGFRcre, can replace creatinine clearance, given that GFRcys is more accurate than eGFRcre in patients with sarcopenia^6^. Thus, Cr × eGFRcys has been recently proposed as a novel serum sarcopenia index^25^.

Given the high mortality risk in patients with CKD experiencing muscle wasting, we hypothesized that these sarcopenia indices, Cr/CysC, eGFRcys/eGFRcre, eGFR_Diff_, and Cr × eGFRcys, may be useful in predicting mortality in patients with non-dialysis CKD. Thus, we conducted a retrospective cohort study including the CKD registry of our hospital to explore the association between sarcopenia indices and overall mortality in patients with non-dialysis CKD.

## Materials and methods

### Setting and participants

This retrospective cohort study was conducted in the CKD outpatient clinic of Hualien Tzu Chi Hospital, a medical center in eastern Taiwan. Since 2008, patients with CKD treated in our hospital have been enrolled in Taiwan renal care program, which provides multidisciplinary care that integrates nephrologists, nurses, and dietitians^26^. Patients with stage 1–4 CKD underwent clinical evaluation every three months, whereas those with stage 5 CKD underwent clinical evaluation every month. For all patients, clinical information, including baseline demographics, CKD etiology and stage, comorbidities, and laboratory data, were established in the CKD registry of Tzu Chi Hospital through face-to-face interviews by trained CKD nurses.

In the present study, all non-dialysis CKD patients (stage 1–5) who visited the outpatient clinic between January 1, 2016 and December 31, 2018 were screened. The study start date was based on the first availability of serum cystatin measurements in our hospital. CKD was defined as a decrease in renal function or the presence of kidney damage for more than three months, according to the Kidney Disease Outcomes Quality Initiative guidelines^27^. Among 1213 patients in the initial screening, those younger than 20 years of age (n = 4), those without serum cystatin C data (n = 41), those who were lost to follow-up for more than six months (n = 14), and those who were misdiagnosed as CKD (n = 13) were excluded. Therefore, 1141 patients were included in the final analysis.

The study was approved by the Research Ethics Committee of Hualien Tzu-Chi Hospital, Buddhist Tzu Chi Medical Foundation (IRB 109-288-B), and all methods were performed in accordance with the relevant guidelines and regulations. Due to the retrospective study design, the requirement of written informed consent was waived by the Research Ethics Committee of Hualien Tzu-Chi Hospital, Buddhist Tzu Chi Medical Foundation (IRB 109-288-B).

### Baseline data collection

Baseline information retrieved from the CKD registry included the following demographic data: age, sex, height, weight, body mass index (BMI), smoking (never, former, or current), exercise (type, duration, and frequency). Regular exercise was defined as moderate-intensity activity ≥ 150 min per week or vigorous-intensity activity ≥ 75 min per week^28^. Comorbid conditions, including diabetes mellitus (DM), chronic glomerulonephritis (GN), hypertension, cardiovascular (CV) disease (ischemic heart disease and congestive heart failure), stroke, and malignancy, were collected from the electrical medical records.

### Measurement of creatinine, cystatin C, and other laboratory data

Baseline blood and urine biochemical data were obtained from the registry. Serum creatinine was measured using an autoanalyzer (Siemens Advia 1800, Siemens Healthcare, Henkestr, Germany), and serum cystatin C levels were measured by a nephelometric immunoassay (Siemens). eGFRcre was calculated using the Modification of Diet in Renal Disease equation as follows: eGFRcre = 186.3 × creatinine^−1.154^ × age^−0.203^ × (0.742, if female)^29^. eGFRcys was calculated using the Chronic Kidney Disease Epidemiology Collaboration (CKD-EPI) cystatin C equation as follows: eGFRcys = 133 × min (cystatin C/0.8, 1)^−0.499^ × max (cystatin C/0.8, 1)^−1.328^ × 0.996^age^ × (0.932, if female), in which min indicates the minimum of cystatin C/0.8 or 1 and max indicates the maximum of cystatin C/0.8 or 1^30^. In this study, CKD staging was based on eGFRcys.

In the present study, the sarcopenia indices were calculated as follows: Cr/CysC = serum Cr (mg/dL)/serum CysC (mg/L); eGFRcys/eGFRcre = eGFRcys (mL/min/1.73 m^2^)/eGFRcre (mL/min/1.73 m^2^); eGFR_Diff_ = eGFRcys − eGFRcre; and Cr × eGFRcys = serum Cr (mg/dL) × eGFRcys (mL/min/1.73 m^2^).

Serum blood urea nitrogen (BUN), albumin, and total cholesterol (TCH) levels and urine protein/creatinine ratio (UPCR) were measured using an autoanalyzer (Siemens Advia 1800).

### Patient follow-up and clinical outcomes

For all patients, the first date of available serum creatinine and cystatin C data for the calculation of sarcopenia indices during the enrolled period was defined as the index date. Patients were followed until death, dialysis initiation, transfer to another hospital, or end of the study (December 1, 2020). Death was confirmed through the electronic medical records of the hospital and outpatient clinic. Out-of-hospital death was confirmed by phone interviews with the family of patients. The primary study outcome was overall mortality. Information regarding the cause of death in the registry was not available, and cause-specific death could not be analyzed in the present study.

### Statistical analysis

Continuous variables were expressed as means ± standard deviation or as medians with interquartile ranges, according to the normality evaluated by the Kolmogorov–Smirnov test. Variables between groups were compared by Student’s independent *t* test or the Mann–Whitney *U* test. Categorical variables were expressed as absolute numbers with relative frequencies and analyzed by the chi-square or Fisher’s exact test.

Due to the presence of missing data for some baseline biochemical parameters including albumin, TCH, hemoglobin, BUN, and UPCR, with rates ranging from 1 to 19%, multiple imputation was performed. Assuming that the data were missing at random, automatic imputation methods in the SPSS statistical software were used. To account for missing values, five complete datasets were generated, each with missing values imputed using linear regression analyses. The parameter estimates from each imputed dataset were averaged to obtain a final set of parameter estimates^31^.

Receiver operating characteristic (ROC) curves were constructed to assess the predictive value of sarcopenia indices for overall mortality. Area under the ROC curve (AUC), cut-off value, sensitivity, specificity, positive predictive value, and negative predictive value were established. The AUCs among different indices were compared using the DeLong test.

For the analysis of overall survival, patients who were transferred to another hospital, those who received dialysis, and those who did not experience mortality during the follow-up period were censored. The Kaplan–Meier analysis with the log-rank test was used to compare overall survival between patients with normal and low sarcopenia indices, who were dichotomized based on sex-specific cut-off values generated from the ROC curves. The Cox proportional hazards models were used to assess the associations of sarcopenia indices with overall mortality. Sarcopenia indices were analyzed as both continuous and categorical variables. In addition to age and sex, adjusted factors in the multivariate model included clinically relevant risk factors as well as variables with significant differences between the survivor and non-survivor groups. Furthermore, two sensitivity analyses were performed. First, the association between sarcopenia indices and mortality was analyzed in 862 patients with complete laboratory data. Second, regarding the potential competing effect of dialysis on the survival analysis, the sub-distribution hazard model was used in which dialysis was treated as a competing event^32^. Finally, subgroup analyses based on age groups (< 65 and ≥ 65 years), sex, DM (yes or no), and CKD stage (1–3 and 4–5) were performed.

All statistical analyses were performed using SPSS (version 19.0; SPSS, Chicago, IL, USA) and SAS (version 9.4; SAS Institute, North Carolina, US). A *p* value of less than 0.05 was considered to indicate statistical significance.

## Results

A total of 1141 CKD patients, with a median age of 71 (62–81) years, were included in the study. The rates of patients with stage 1–2, 3, 4, and 5 CKD were 8.3%, 33.7%, 37.1%, and 20.9%, respectively. Table 1 summarizes the demographic data and clinical characteristics of all participants and those stratified by sex. In the overall cohort, there were 664 (58.2%) male patients and 477 (41.8%) female patients. In addition, 623 (54.6%), 400 (35.1%), 875 (76.7%), 248 (21.7%), 123 (10.8%), and 91 (8.0%) patients had DM, chronic GN, hypertension, CV disease, stroke, and malignancy, respectively. The rates of stroke, smoking, and regular exercise were significantly higher in male patients than in female patients (*p* = 0.03, *p* < 0.001, and *p* = 0.01, respectively). Regarding the laboratory data, male patients had higher hemoglobin (*p* < 0.001), creatinine (*p* < 0.001), and eGFRcre (*p* = 0.01) values and lower TCH (*p* < 0.001) and UPCR (*p* = 0.01) values compared to female patients. In addition, all four sarcopenia indices were significantly different between the sexes, with Cr/CysC and Cr × eGFRcys higher in male patients (*p* < 0.001), while eGFRcys/eGFRcre and eGFR_Diff_ lower in male patients (*p* < 0.001 and *p* = 0.01, respectively).

**Table 1:** Demographic and clinical characteristics of study population.

Characteristics	All patients (*n* = 1141)	Male (*n* = 664)	Female (*n* = 477)	*p*
Age (years)	71 (62–81)	71 (62–82)	73 (63–80)	0.80
**Diseases, n (%)**				
DM	623 (54.6)	351 (52.9)	272 (57.0)	0.16
Chronic GN	400 (35.1)	230 (34.6)	170 (35.6)	0.73
Hypertension	875 (76.7)	507 (76.4)	368 (77.1)	0.76
CV disease	248 (21.7)	132 (19.9)	116 (24.3)	0.07
Stroke	123 (10.8)	83 (12.5)	40 (8.4)	0.03*
Malignancy	91 (8.0)	50 (7.5)	41 (8.6)	0.51
**Smoking status, n (%)**				
Never-smokers	830 (72.7)	389 (58.6)	441 (92.5)	< 0.001*
Ex-smokers	148 (13.0)	134 (20.2)	14 (2.9)	
Current smokers	163 (14.3)	141 (21.2)	22 (4.6)	
Regular exercise, n (%)	363 (31.8)	232 (34.9)	131 (27.5)	0.01*
BMI (kg/m^2^)	25.5 (22.8–28.5)	25.5 (23.0–28.3)	25.4 (22.5–28.6)	0.80
**Laboratory data**				
Hemoglobin (g/dL)	11.4 (9.9–12.9)	12.0 (10.3–13.5)	10.9 (9.6–12.0)	< 0.001*
Albumin (g/dL)	4.0 (3.8–4.2)	4.0 (3.8–4.3)	4.0 (3.8–4.2)	0.63
TCH (mg/dL)	161 (139–187)	158 (137–182)	168 (142–192)	< 0.001*
Glucose (mg/dL)	108 (92–141)	107 (92–134)	110 (91–150)	0.20
BUN (mg/dL)	33 (24–50)	33 (24–49)	34 (23–52)	0.98
Creatinine (mg/dL)	2.0 (1.4–3.1)	2.1 (1.6–3.2)	1.8 (1.2–3.0)	< 0.001*
eGFRcre (mL/min/1.73 m^2^)	32 (18–46)	33 (21–47)	29 (17–45)	0.01*
Cystatin C (mg/L)	2.1 (1.6–3.0)	2.1 (1.6–3.0)	2.1 (1.5–3.2)	0.97
eGFRcys (mL/min/1.73 m^2^)	27 (16–39)	27 (18–38)	25 (15–39)	0.08
UPCR (g/g)	0.9 (0.2–2.7)	0.8 (0.2–2.6)	1.1 (0.3–2.8)	0.01*
**Sarcopenia indices**				
Cr/CysC	0.98 (0.83–1.15)	1.05 (0.90–1.21)	0.89 (0.76–1.04)	< 0.001*
eGFRcys/eGFRcre	0.89 (0.74–1.07)	0.88 (0.72–1.14)	0.92 (0.76–1.09)	< 0.001*
eGFR_Diff_	− 2.9 (− 9.0 to 1.3)	− 3.5 (− 9.7 to 1.0)	− 2.1 (− 7.6 to 1.9)	0.01*
Cr × eGFRcys	55.6 (45.4–67.0)	60.3 (50.7–72.1)	48.5 (40.5–58.6)	< 0.001*

Values for continuous variables are given as means ± standard deviations or medians and interquartile ranges. Categorical variables are expressed as numbers (%).

*DM* diabetes mellitus, *GN* glomerulonephritis, *CV* cardiovascular, *BMI* body mass index, *TCH* total cholesterol, *BUN* blood urea nitrogen, *eGFRcre* estimated glomerular filtration rate from serum creatinine, *eGFRcys* estimated glomerular filtration rate from serum cystatin C, *UPCR* urine protein/creatinine ratio, *eGFR*_*Diff*_ eGFRcys − eGFRcre.

*p < 0.05 was considered statistically significant between male and female patients.

During a median follow-up of 39 months, 116 (10.2%) patients died. Table 2 shows the comparison of clinical characteristics between the survivors and non-survivors. The patients were older (*p* < 0.001), and the prevalence rates for CV disease (*p* = 0.01) and malignancy (*p* < 0.001) were higher in the non-survivor group compared with the survivor group. In addition, the non-survivors had lower BMI (*p* = 0.03), serum hemoglobin (*p* < 0.001), albumin (*p* < 0.001), TCH (*p* < 0.02), eGFRcre (*p* = 0.01), and eGFRcys (*p* < 0.001) and higher serum creatinine (*p* = 0.03) and cystatin C (*p* < 0.001) compared with the survivors. Notably, all four sarcopenia indices, Cr/CysC, eGFRcys/eGFRcre, eGFR_Diff_, and Cr × eGFRcys, were significantly lower in the non-survivor group than in the survivor group (*p* < 0.001 for all).

**Table 2:** Comparisons of clinical characteristics between the survivors and non-survivors.

Characteristics	Survivors (*n* = 1025)	Non-survivors (*n* = 116)	*p*
Age (years)	70 (61–80)	82 (72–88)	< 0.001*
Gender (female)	430 (42.0)	47 (40.5)	0.77
**Diseases, n (%)**			
DM	551 (53.8)	72 (62.1)	0.09
Chronic GN	362 (35.3)	38 (32.8)	0.58
Hypertension	786 (76.7)	89 (76.7)	0.99
CV disease	211 (20.6)	37 (31.9)	0.01*
Stroke	107 (10.4)	16 (13.8)	0.27
Malignancy	72 (7.0)	19 (16.4)	< 0.001*
**Smoking status, n (%)**			
Never-smokers	750 (73.2)	80 (69.0)	0.57
Ex-smokers	132 (12.9)	16 (13.8)	
Current smokers	143 (14.0)	20 (17.2)	
Regular exercise, n (%)	329 (32.1)	34 (29.3)	0.54
BMI (kg/m^2^)	25.6 (22.9–28.6)	24.5 (21.9–28.0)	0.03*
**Laboratory data**			
Hemoglobin (g/dL)	11.4 (10.1–13.0)	10.7 (8.9–12.1)	< 0.001*
Albumin (g/dL)	4.0 (3.8–4.3)	3.9 (3.6–4.1)	< 0.001*
TCH (mg/dL)	163 (140–188)	156 (136–179)	0.02*
Glucose (mg/dL)	108 (91–139)	108 (92–152)	0.59
BUN (mg/dL)	33 (23–49)	40 (28–59)	0.002*
Creatinine (mg/dL)	2.0 (1.4–3.0)	2.3 (1.6–3.4)	0.03*
eGFRcre (mL/min/1.73 m^2^)	32 (19–47)	28 (16–41)	0.01*
Cystatin C (mg/L)	2.1 (1.5–3.0)	2.6 (2.0–3.7)	< 0.001*
eGFRcys (mL/min/1.73 m^2^)	28 (17–40)	20 (13–28)	< 0.001*
UPCR (g/g)	0.9 (0.2–2.7)	1.0 (0.3–2.4)	0.86
**Sarcopenia indices**			
Cr/CysC	1.00 (0.84–1.17)	0.86 (0.76–1.01)	< 0.001*
eGFRcys/eGFRcre	0.91 (0.77–1.08)	0.75 (0.63–0.87)	< 0.001*
eGFR_Diff_	− 2.4 (− 8.5 to 1.7)	− 6.1 (− 11.5 to − 2.5)	< 0.001*
Cr × eGFRcys	57.1 (46.4–68.3)	44.8 (37.7–53.2)	< 0.001*

Values for continuous variables are given as means ± standard deviations or medians and interquartile ranges. Categorical variables are expressed as numbers (%).

*DM* diabetes mellitus, *GN* glomerulonephritis, *CV* cardiovascular, *BMI* body mass index, *TCH* total cholesterol, *BUN* blood urea nitrogen, *eGFRcre* estimated glomerular filtration rate from serum creatinine, *eGFRcys* estimated glomerular filtration rate from serum cystatin C, *UPCR* urine protein/creatinine ratio, *eGFR*_*Diff*_ eGFRcys − eGFRcre.

*p < 0.05 was considered statistically significant between the survivors and non-survivors.

**Figure 1:**
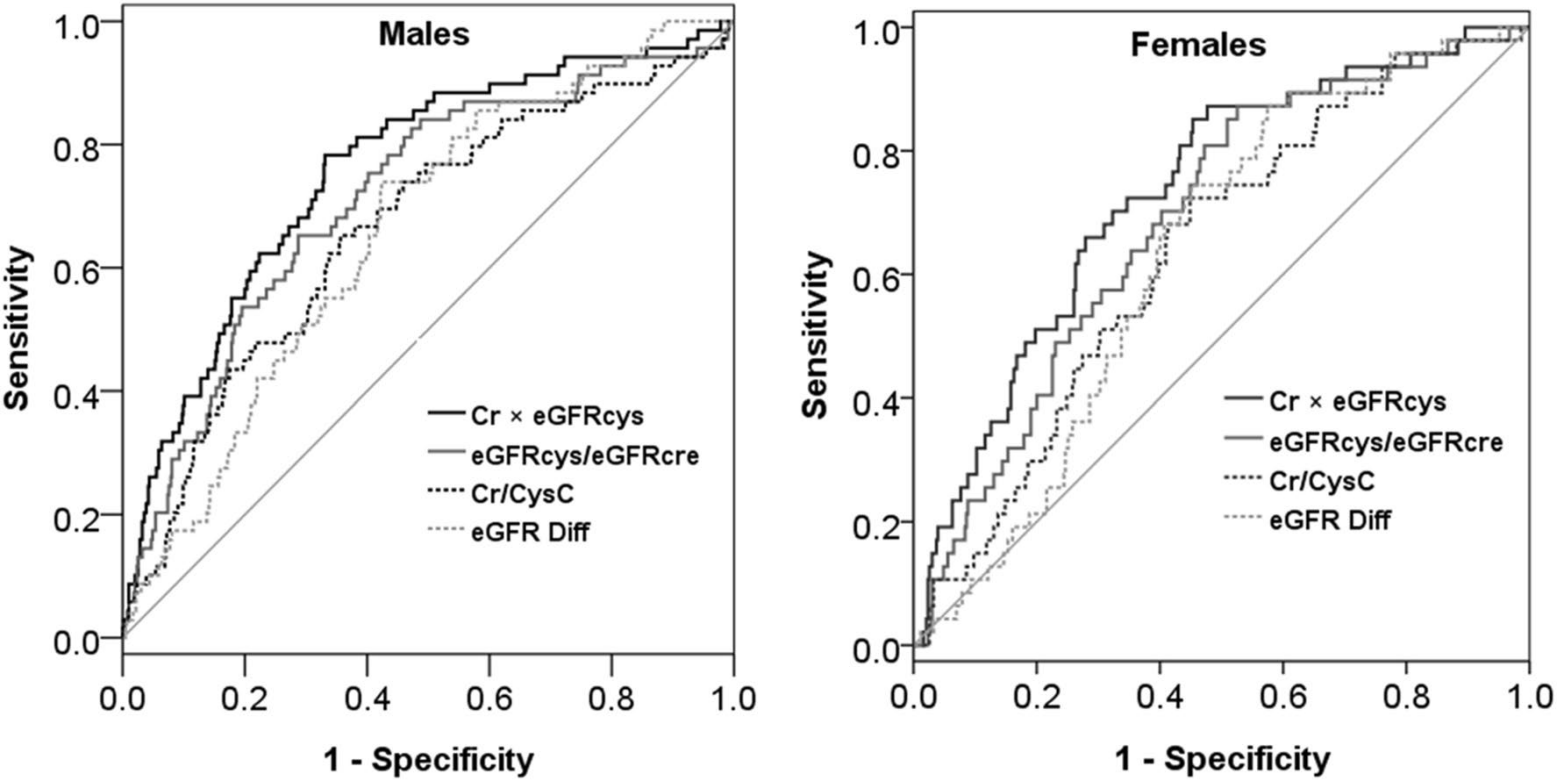
Receiver operating characteristic curves of sarcopenia indices on the prediction of mortality in male and female CKD patients.

The comparison of ROC curves for sarcopenia indices for the prediction of mortality is shown in Fig. 1 and Table 3. All four sarcopenia indices had acceptable discriminative power in both sexes. Specifically, Cr × eGFRcys had the best predictive power (AUC 0.758, 95% confidence interval [CI] 0.723–0.790 for males; AUC 0.732, 95% CI 0.690–0.771 for females), followed by eGFRcys/eGFRcre (AUC 0.714, 95% CI 0.678–0.748 for males; AUC 0.688, 95% CI 0.644–0.729 for females), Cr/CysC (AUC 0.665, 95% CI 0.628–0.701 for males; AUC 0.638, 95% CI 0.593–0.681 for females) and eGFR_Diff_ (AUC 0.659, 95% CI 0.622–0.695 for males; AUC 0.632, 95% CI 0.587–0.675 for females). Pairwise comparisons of AUC among four indices in both gender were all highly significant (p < 0.001) except for the comparison between Cr/CysC and eGFR_Diff_. The optimal cut-off value, sensitivity, specificity, positive predictive value, and negative predictive value to predict mortality for the four indices are shown in Table 3.

**Table 3:** Predictive validity of sarcopenia indices on overall mortality, overall and stratified by gender.

	AUC (95% CI)	Cut-off	Sen (%)	Spe (%)	PPV (%)	NPV (%)
**Low Cr/CysC**						
Overall	0.647 (0.619–0.675)*					
Male	0.665 (0.628–0.701)*	0.98	65.2	64.4	17.5	94.1
Female	0.638 (0.593–0.681)*	0.88	72.3	55.1	15.0	94.8
**Low eGFRcys/eGFRcre**						
Overall	0.702 (0.674–0.728)*					
Male	0.714 (0.678–0.748)*	0.83	65.2	71.3	20.8	94.6
Female	0.688 (0.644–0.729)*	1.01	87.2	47.4	15.4	97.1
**Low eGFR**_**Diff**_						
Overall	0.646 (0.618–0.674)*					
Male	0.659 (0.622–0.695)*	− 4.2	73.9	57.7	16.8	95.0
Female	0.632 (0.587–0.675)*	− 0.6	87.2	42.6	14.2	96.8
**Low Cr × eGFRcys**						
Overall	0.728 (0.701–0.753)*					
Male	0.758 (0.723–0.790)*	56.3	78.3	66.9	21.5	96.4
Female	0.732 (0.690–0.771)*	48.3	85.1	54.7	17.0	97.1

*AUC* area under curves, *CI* confidence interval, *Sen* sensitivity, *Spe* specificity, *PPV* positive predictive value, *NPV* negative predictive value.

*p < 0.05 was considered statistically significant.

**Figure 2:**
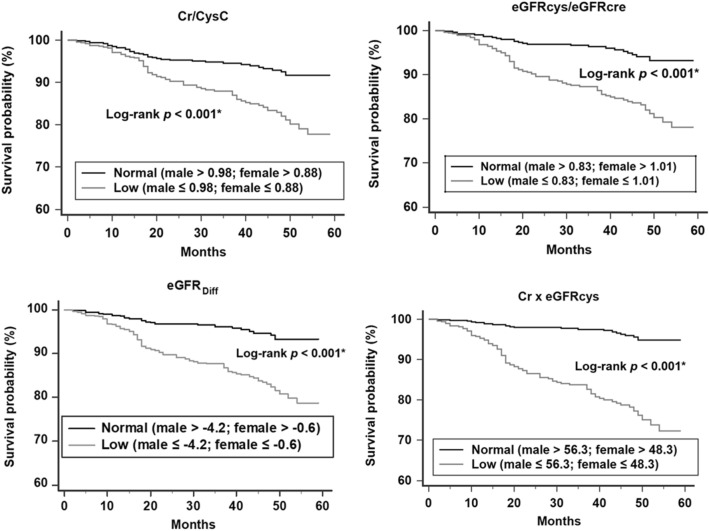
Kaplan–Meier survival analysis of study population with normal or low sarcopenia indices.

As shown in Fig. 2, there were significant differences in survival rates between the patients with low and normal sarcopenia indices (*p* < 0.001 for all four) after stratification according to the sex-specific cut-off values for predicting mortality.

The association between overall mortality and sarcopenia indices using univariate and multivariate Cox proportional hazards models are shown in Table 4. In unadjusted models, Cr/CysC, eGFRcys/eGFRcre, eGFR_Diff_, and Cr × eGFRcys were associated with overall mortality, as both continuous or categorical variables. These associations remained significant after adjusting for age, sex, BMI, DM, CV disease, malignancy, hemoglobin, albumin, TCH, BUN, and eGFRcys. Similar results were found with two separate sensitivity analyses, one including only 862 patients with complete data and the other using a sub-distribution hazard model (Supplementary Tables S1 and S2).

**Table 4:** Hazard ratios for death according to the sarcopenia indices, analyzed as a continuous or categorical variable.

Variable		Hazard ratio (95% CI)		
		Unadjusted	Model 1	Model 2
Cr/CysC	Per 1 SD increase	0.66 (0.52–0.84)*	0.72 (0.55–0.93)*	0.68 (0.53–0.87)*
	Low (male ≤ 0.98; female ≤ 0.88)^a^	2.58 (1.75–3.81)*	2.20 (1.48–3.28)*	2.29 (1.53–3.42)*
	Q4 (> 1.15)	1 (reference)	1 (reference)	1 (reference)
	Q3 (0.98–1.15)	1.52 (0.76–3.06)	1.39 (0.69–2.80)	1.78 (0.87–3.61)
	Q2 (0.83–0.97)	2.26 (1.17–4.38)*	2.01 (1.02–3.93)*	2.57 (1.31–5.03)*
	Q1 (< 0.83)	3.31 (1.76–6.23)*	2.85 (1.47–5.54)*	3.44 (1.76–6.72)*
eGFRcys/eGFRcre	Per 1 SD increase	0.47 (0.37–0.61)*	0.55 (0.43–0.72)*	0.67 (0.52–0.85)*
	Low (male ≤ 0.83; female ≤ 1.01)^a^	3.48 (2.24–5.43)*	2.95 (1.86–4.67)*	2.46 (1.55–3.91)*
	Q4 (> 1.07)	1 (reference)	1 (reference)	1 (reference)
	Q3 (0.89–1.07)	1.39 (0.63–3.10)	1.26 (0.56–2.80)	1.38 (0.61–3.09)
	Q2 (0.75–0.88)	3.07 (1.52–6.24)*	2.37 (1.16–4.86)*	2.35 (1.14–4.85)*
	Q1 (< 0.75)	5.82 (2.97–11.39)*	4.32 (2.17–8.57)*	3.26 (1.62–6.55)*
eGFR_Diff_	Per 1 SD increase	0.83 (0.73–0.93)*	0.78 (0.66–0.92)*	0.63 (0.51–0.79)*
	Low (male ≤ − 4.2; female ≤ − 0.6)^a^	3.39 (2.17–5.32)*	2.81 (1.77–4.47)*	2.88 (1.80–4.60)*
	Q4 (> 1.34)	1 (reference)	1 (reference)	1 (reference)
	Q3 (− 2.9 to 1.34)	2.05 (0.98–4.28)	1.79 (0.85–3.74)	1.23 (0.54–2.38)
	Q2 (− 9.0 to − 2.9)	3.77 (1.95–7.30)*	3.00 (1.54–5.84)*	2.18 (1.11–4.27)*
	Q1 (< − 9.0)	3.53 (1.81–6.86)*	2.61 (1.32–5.16)*	2.58 (1.28–5.16)*
Cr × eGFRcys	Per 1 SD increase	0.39 (0.30–0.49)*	0.39 (0.30–0.52)*	0.58 (0.43–0.78)*
	Low (male ≤ 56.3; female ≤ 48.3)^a^	6.37 (4.01–10.15)*	5.13 (3.16–8.32)*	3.34 (2.04–5.47)*
	Q4 (> 67.0)	1 (reference)	1 (reference)	1 (reference)
	Q3 (55.6–67.0)	1.54 (0.67–3.56)	1.47 (0.63–3.41)	1.10 (0.47–2.57)
	Q2 (45.5–55.5)	3.53 (1.69–7.40)*	3.15 (1.47–6.74)*	2.01 (0.93–4.33)
	Q1 (< 45.5)	7.68 (3.81–15.46)*	7.18 (3.37–15.28)*	3.19 (1.46–6.96)*

Model 1: adjusted for age, sex, BMI, DM, CV disease, and malignancy. Model 2: Model 1 + hemoglobin, albumin, TCH, BUN, and eGFRcys.

*CI* confidence interval, *SD* standard deviation, *Q* quartile.

*p < 0.05 was considered statistically significant.

^a^Normal group as reference.

**Figure 3:**
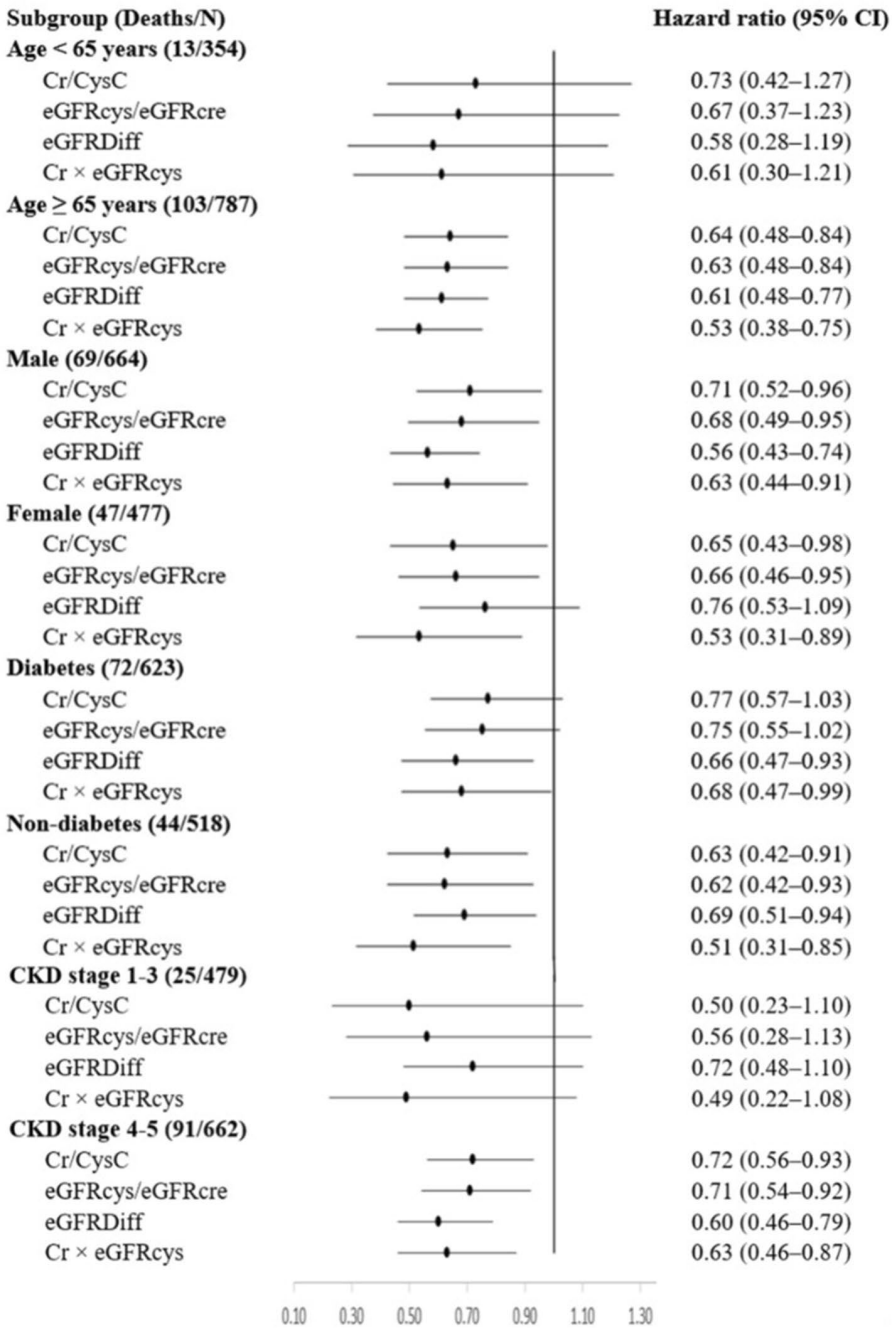
Adjusted hazard of mortality associated with each 1-SD increase in sarcopenia indices, by subgroup. Model fully adjusted for age, sex, BMI, DM, CV disease, malignancy, hemoglobin, albumin, TCH, BUN, and eGFRcys.

In subgroup analysis, the trend association between low sarcopenia indices and increased mortality was consistent across different subgroups (Fig. 3).

## Discussion

This is the first study to report the association between the four sarcopenia indices, Cr/CysC, eGFRcys/eGFRcre, eGFR_Diff_, and Cr × eGFRcys, and overall mortality in patients with non-dialysis CKD. Our analyses revealed that all four indices independently predicted mortality in patients with non-dialysis. Among the evaluated indices, Cr × eGFRcys outperformed Cr/CysC, eGFRcys/eGFRcre, and eGFR_Diff_ in predicting mortality in both sexes.

Emerging evidence suggests the association between sarcopenia indices and clinical outcomes in various populations. A low Cr/CysC was shown to be associated with increased risk of bone fractures in patients with type 2 DM^33^ and to predict long-term functional disability in neurocritically ill patients^34^; it was also reported to contribute to higher mortality in adult patients in the intensive care unit^10,35^ and senior patients^9,18^. In osteoporotic women, a lower eGFRcys/eGFRcre was associated with bone fractures^20^. In a large cohort of hypertensive participants, eGFR_Diff_ is shown to be associated with frailty and adverse outcomes, including falls, hospitalizations, CV events, and mortality^21^. The newly proposed index, Cr × eGFRcys, has been recently shown to predict surgical complications in colorectal cancer^36^. Consistent with these studies, we showed a significant association between these four sarcopenia indices and overall mortality in CKD, which was independent of the well-known risk factors.

There is a paucity of studies comparing several sarcopenia indices together. The present study findings suggest that Cr × eGFRcys may be superior to the other three indices for predicting mortality. Consistent with these findings, two recent studies showed that the correlation of Cr × eGFRcys with skeletal muscle mass and handgrip strength was better than that of Cr/CysC in patients with cancer^16,36^. We speculate that, compared to the other three indices, Cr × eGFRcys might be more closely correlated with timed urinary creatinine excretion, a well-established and reliable urinary marker for estimating muscle wasting and predicting mortality in patients with CKD^23^. Unfortunately, 24-h urine samples were not available in the present study and future studies are necessary to confirm this hypothesis.

Regarding the modest correlations observed between sarcopenia indices, skeletal muscle mass, and strength in previous studies, other factors beyond skeletal muscle wasting might explain the association between low sarcopenia indices and mortality in the present study. For example, malignancy is associated with increased serum cystatin C levels^37^, while CV disease is linked to lower creatinine levels^38^. In our study, the association between the sarcopenia indices and mortality remains unchanged after adjusting these two factors in the models. However, some other factors affecting serum creatinine or cystatin C were not collected in this study. Low dietary intake, fluid overload, and chronic liver disease lower serum creatinine levels^25,39^; hyperthyroidism and corticosteroid use increase serum cystatin C levels^40,41^; inflammation and obesity reduce serum creatinine while simultaneously elevating cystatin C levels^6^. These unmeasured factors might further magnify the impact of sarcopenia indices on mortality.

Currently, the diagnostic workup and cut-off values for sarcopenia are well-established in older populations^42–45^ but not in patients with CKD. The reported prevalence of sarcopenia in patients with non-dialysis CKD ranges widely from 5.9 to 41.4% using different criteria^4,46,47^, suggesting that the best diagnostic criteria for sarcopenia in CKD remain unclear. The mortality-based cut-off values for sarcopenia indices used in the present study could provide a novel insight on real-world clinical practices to identify patients with CKD who are at increased risk of mortality.

To our knowledge, this is the first study to report the association between low sarcopenia indices based on serum creatinine and cystatin C and mortality in patients with non-dialysis CKD using four different indices simultaneously in a real-world clinical setting. However, several limitations should be acknowledged. First, this was a retrospective cohort study and the causal relationship should be cautiously interpreted. Second, skeletal muscle mass and strength, as well as gold-standard measure of renal function, were not available in our CKD cohort. Third, inflammatory markers, such as C-reactive protein and interleukin-6, were not measured. Fourth, the etiologies of mortality could not be clarified in the present study. Fifth, we did not exclude patients with fluctuating renal function, in which creatinine and cystatin C are not in a steady state and their ratio may be less correlated with skeletal muscle status. However, we believe that most patients in the CKD outpatient clinic had a relatively stable renal function. Interestingly, a recent study showed that low Cr/CysC was associated with mortality even in patients with acute kidney injury undergoing continuous kidney replacement therapy in the intensive care unit^48^. Finally, the study patients were recruited from the outpatient clinic of a single medical center in Taiwan and the study findings may not be generalized to other ethnic populations or different clinical settings.

## Conclusion

All four sarcopenia indices based on serum creatinine and cystatin C, which are simple and widely available in the clinical setting, predicted overall mortality independently of well-established risk factors in patients with non-dialysis CKD. These findings have important clinical implications. Beyond their traditional use as renal function estimates, these indices provide important prognostic information and may be considered as promising surrogate markers for sarcopenia in patients with non-dialysis CKD. However, further studies are needed to establish the diagnostic validity of these sarcopenia indices.

## Supplementary Information

Supplementary Tables.

## Data Availability

The data underlying this article will be shared on reasonable request to the corresponding author.
