# Supplementary material for: Serum indices based on creatinine and cystatin C predict mortality in patients with non-dialysis chronic kidney disease
Source: Sci Rep. 2021 Aug 19;11:16863. doi: 10.1038/s41598-021-96447-9 (PMC8377030; doi:10.1038/s41598-021-96447-9)
Supplement: Supplementary file 1 — Supplementary Tables. [file 41598_2021_96447_MOESM1_ESM.docx]

**Table S1.** Hazard ratios for death according to the sarcopenia indices among 862 patients with complete data. Model 1: adjusted for age, sex, BMI, DM, CV disease, and malignancy.

Model 2: Model 1 + hemoglobin, albumin, cholesterol, BUN, and eGFRcys.

*p <.05 was considered statistically significant. CI, confidence interval; SD, standard deviation; Q, quartile.

| **Variable** | | **Hazard ratio (95% CI)** | | |
| --- | --- | --- | --- | --- |
|  |  | **Unadjusted** | **Model 1** | **Model 2** |
| Cr/CysC | Per 1 SD increase | 0.58 (0.43–0.77)* | 0.65 (0.47–0.89)* | 0.58 (0.42–0.79)* |
|  | Low (male ≤ 0.98; female ≤ 0.88) | 2.90 (1.85–4.55)* | 2.35 (1.48–3.74)* | 2.69 (1.68–4.31)* |
|  | Q4 (>1.15) | 1 (reference) | 1 (reference) | 1 (reference) |
|  | Q3 (0.98–1.15) | 1.32 (0.58–3.01) | 1.15 (0.50–2.63) | 1.49 (0.64–3.45) |
|  | Q2 (0.83–0.97) | 2.47 (1.15–5.31)* | 1.98 (0.91–4.33)* | 2.78 (1.27–6.10)* |
|  | Q1 (<0.83) | 3.91 (1.89–8.07)* | 3.04 (1.41–6.54)* | 4.18 (1.92–9.10)* |
| eGFRcys/eGFRcre | Per 1 SD increase | 0.42 (0.32–0.57)* | 0.55 (0.43–0.71)* | 0.57 (0.42–0.78)* |
|  | Low (male ≤ 0.83; female ≤ 1.01) | 3.65 (2.22–6.02)* | 2.81 (1.66–4.75)* | 2.51 (1.48–4.26)* |
|  | Q4 (>1.07) | 1 (reference) | 1 (reference) | 1 (reference) |
|  | Q3 (0.89–1.07) | 1.23 (0.48–3.11) | 1.12 (0.44–2.84) | 1.14 (0.44–2.92) |
|  | Q2 (0.75–0.88) | 3.12 (1.41–6.92)* | 2.25 (1.00–5.05)* | 2.26 (1.00–5.11) |
|  | Q1 (<0.75) | 6.37 (3.00–13.54)* | 4.55 (2.10–9.87)* | 3.69 (1.66–8.21)* |
| eGFR_Diff_ | Per 1 SD increase | 0.83 (0.73–0.95)* | 0.78 (0.63–0.96)* | 0.65 (0.49–0.77)* |
|  | Low (male ≤ -4.2; female ≤ -0.6) | 3.45 (2.07–5.73)* | 2.60 (1.54–4.42)* | 2.90 (1.69–4.99)* |
|  | Q4 (>1.34) | 1 (reference) | 1 (reference) | 1 (reference) |
|  | Q3 (-2.9–1.34) | 1.78 (0.76–4.16) | 1.49 (0.64–3.51) | 0.82 (0.34–1.97) |
|  | Q2 (-9.0– -2.9) | 3.89 (1.86–8.13)* | 2.96 (1.40–6.24)* | 2.19 (1.03–4.67)* |
|  | Q1 (< -9.0) | 3.74 (1.78–7.83)* | 2.59 (1.21–5.54)* | 2.52 (1.14–5.57)* |
| Cr × eGFRcys | Per 1 SD increase | 0.34 (0.25–0.46)* | 0.37 (0.26–0.52)* | 0.50 (0.35–0.73)* |
|  | Low (male ≤ 56.3; female ≤ 48.3) | 6.60 (3.93–11.10)* | 4.89 (2.83–8.47)* | 3.39 (1.95–5.91)* |
|  | Q4 (>67.0) | 1 (reference) | 1 (reference) | 1 (reference) |
|  | Q3 (55.6–67.0) | 1.61 (0.62–4.14) | 1.42 (0.55–3.69) | 0.96 (0.37–2.53) |
|  | Q2 (45.5–55.5) | 4.28 (1.86–9.85)* | 3.39 (1.43–8.05)* | 2.01 (0.84–4.82) |
|  | Q1 (<45.5) | 8.23 (3.70–18.31)* | 6.52 (2.70–15.71)* | 2.93 (1.17–7.29)* |

**Table S2.** Sub-distrubition hazard ratios for death according to the sarcopenia indices as a continuous or categorical variable. Model 1: adjusted for age, sex, BMI, DM, CV disease, and malignancy. Model 2: Model 1 + hemoglobin, albumin, cholesterol, BUN, and eGFRcys.

*p <.05 was considered statistically significant. CI, confidence interval; SD, standard deviation; Q, quartile.

| **Variable** | | **Hazard ratio (95% CI)** | | |
| --- | --- | --- | --- | --- |
|  |  | **Unadjusted** | **Model 1** | **Model 2** |
| Cr/CysC | Per 1 SD increase | 0.60 (0.46–0.79)* | 0.65 (0.48–0.89)* | 0.60 (0.45–0.79)* |
|  | Low (male ≤ 0.98; female ≤ 0.88) | 3.04 (2.06–4.49)* | 2.52 (1.68–3.77)* | 3.01 (1.98–4.58)* |
|  | Q4 (>1.15) | 1 (reference) | 1 (reference) | 1 (reference) |
|  | Q3 (0.98–1.15) | 2.00 (0.99–4.02) | 1.75 (0.87–3.53) | 2.51 (1.21–5.20)* |
|  | Q2 (0.83–0.97) | 2.99 (1.55–5.80)* | 2.61 (1.32–5.16)* | 3.91 (1.98–7.74)* |
|  | Q1 (<0.83) | 4.49 (2.38–8.45)* | 3.73 (1.90–7.30)* | 5.30 (2.63–10.68)* |
| eGFRcys/eGFRcre | Per 1 SD increase | 0.45 (0.34–0.60)* | 0.54 (0.40–0.72)* | 0.59 (0.45–0.77)* |
|  | Low (male ≤ 0.83; female ≤ 1.01) | 3.84 (2.47–5.98)* | 3.19 (1.99–5.11)* | 3.02 (1.86–4.88)* |
|  | Q4 (>1.07) | 1 (reference) | 1 (reference) | 1 (reference) |
|  | Q3 (0.89–1.07) | 1.55 (0.70–3.45) | 1.39 (0.62–3.10) | 1.58 (0.70–3.56) |
|  | Q2 (0.75–0.88) | 3.60 (1.77–7.32)* | 2.72 (1.31–5.63)* | 2.99 (1.43–6.24)* |
|  | Q1 (<0.75) | 6.89 (3.51–13.51)* | 4.94 (2.46–9.92)* | 4.63 (2.28–9.41)* |
| eGFR_Diff_ | Per 1 SD increase | 0.80 (0.73–0.89)* | 0.75 (0.66–0.85)* | 0.62 (0.51–0.75)* |
|  | Low (male ≤ -4.2; female ≤ -0.6) | 3.94 (2.51–6.18)* | 3.20 (2.01–5.11)* | 3.77 (2.32–6.13)* |
|  | Q4 (>1.34) | 1 (reference) | 1 (reference) | 1 (reference) |
|  | Q3 (-2.9–1.34) | 1.96 (0.94–4.09) | 1.72 (0.82–3.60) | 1.29 (0.61–2.70) |
|  | Q2 (-9.0– -2.9) | 4.34 (2.24–8.42)* | 3.39 (1.74–6.60)* | 3.20 (1.61–6.36)* |
|  | Q1 (< -9.0) | 4.24 (2.18–8.26)* | 3.02 (1.52–6.00)* | 3.63 (1.73–7.62)* |
| Cr × eGFRcys | Per 1 SD increase | 0.39 (0.30–0.50)* | 0.40 (0.29–0.54)* | 0.50 (0.36–0.70)* |
|  | Low (male ≤ 56.3; female ≤ 48.3) | 6.58 (4.14–10.46)* | 5.15 (3.14–8.44)* | 3.96 (2.40–6.52)* |
|  | Q4 (>67.0) | 1 (reference) | 1 (reference) | 1 (reference) |
|  | Q3 (55.6–67.0) | 1.60 (0.69–3.71) | 1.51 (0.64–3.55) | 1.28 (0.54–3.02) |
|  | Q2 (45.5–55.5) | 3.86 (1.84–8.13)* | 3.38 (1.53–7.45)* | 2.65 (1.19–5.89)* |
|  | Q1 (<45.5) | 8.08 (3.98–16.39)* | 7.45 (3.35–16.56)* | 4.70 (2.06–10.68)* |
